# Outcomes of minimally invasive isolated tricuspid valve reoperation after left-side valve surgery: A single-center experience

**DOI:** 10.3389/fcvm.2023.1033489

**Published:** 2023-02-01

**Authors:** Jian Liu, Tong Tan, Huanlei Huang, Wenda Gu, Xin Zang, Jianrui Ma, Hongxiang Wu, Haozhong Liu, Jian Zhuang, Jimei Chen, Huiming Guo

**Affiliations:** ^1^Department of Cardiovascular Surgery, Guangdong Cardiovascular Institute, Guangdong Provincial People’s Hospital, Guangdong Academy of Medical Sciences, Southern Medical University, Guangzhou, China; ^2^Guangdong Provincial Key Laboratory of South China Structural Heart Disease, Guangzhou, China; ^3^Shantou University Medical College, Shantou, China

**Keywords:** minimally invasive, tricuspid regurgitation, isolated tricuspid valve reoperation, transcatheter tricuspid valve replacement, ventricular dysfunction

## Abstract

**Background:**

Late severe tricuspid regurgitation (TR) after left-side valve surgery (LSVS) is not uncommon. However, the tricuspid valve has been deemed the forgotten valve because the isolated TR is well tolerated with medication, and reoperation has a higher rate of adverse events. With the advancement of minimally invasive techniques, isolated tricuspid valve reoperation (ITVR) *via* totally endoscopy or transcatheter approach brings the tricuspid valve into spotlight. Our aim is to report the safety and efficacy of minimally invasive ITVR using endoscopic and transcatheter approaches.

**Methods:**

From October 2020 to October 2021, 21 patients with LSVS history and secondary massive TR underwent minimally invasive ITVR in our institution. Baseline characteristics, surgical outcomes and follow-up results were analyzed, and data between the totally endoscopy approach and the transcatheter approach were compared.

**Results:**

Of the 21 cases, totally endoscopic isolated tricuspid valve surgery (EITVS) accounts for 16 (76.2%) cases, with 14 tricuspid valvuloplasty cases, and 2 tricuspid valve replacement cases; the remaining 5 (23.8%) cases underwent transcatheter tricuspid valve replacement (TTVR). The mean age was (60.0 ± 8.4) years, with 15 (71.4%) being female. Minimally invasive ITVR procedures were 100% successfully performed in all patients without any perioperative mortality, sternotomy conversion, or reoperation. During the median follow-up of 16.8 months (IQR, 13.0–20.6 months), New York Heart Association Class improved significantly from baseline (*P* = 0.004). TR severity was significantly improved during postoperative and follow-up period (both *P* < 0.001). Compared with the EITVS group, the TTVR group had a higher clinical risk score [8.00 (8.00, 9.00) vs. 5.00 (3.25, 5.00), *P* = 0.001], but a higher success rate in reducing TR to less than grade 1+ (100 vs. 43.8%, *P* = 0.045) at follow-up.

**Conclusion:**

In our series, minimally invasive ITVR, including EITVS and TTVR, is a safe and feasible option for severe TR after LSVS, and presents excellent early outcomes in selected patients. TTVR is a reliable alternative for patients with high surgical risk. To improve the results of ITVR, it is necessary to improve patient’s preoperative status or perform reoperation before the onset of significant right heart failure. Further studies with a larger sample size and a longer follow-up period are awaited.

## 1. Introduction

Tricuspid regurgitation (TR) is a common valvular heart disease, with a prevalence of more than 70 million people worldwide (1, 2). Secondary or functional TR is the primary etiology of TR, which is usually associated with left-sided valve pathology, right ventricular dilatation or chronic atrial fibrillation. Current guidelines and studies recommend concomitant tricuspid annuloplasty during left-side valve surgery (LSVS) to further reduce the frequent progression of severe TR (3–5). Nevertheless, late severe TR after LSVS remains a major cause of heart failure and mortality (6, 7). The timing of reoperation is challenging as isolated TR is well tolerated by medication and reoperation has a higher rate of adverse events. Some studies have reported that isolated tricuspid valve reoperation (ITVR) using minimally invasive approaches including right mini-thoracotomy and totally endoscopy has achieved encouraging results. Since the first successful case series of LuX-Valve in severe TR has been reported, transcatheter tricuspid valve replacement (TTVR) becomes a novel minimally invasive approach for ITVR (8). Here, we report our experience with minimally invasive ITVR in our institution. The aim of this study is to report the safety and efficacy of minimally invasive ITVR with endoscopic and transcatheter approaches.

## 2. Materials and methods

### 2.1. Study population

From October 2020 to October 2021, 21 consecutive patients who underwent minimally invasive ITVR in our institution were included into this study. The study population was divided into two groups according to the different surgical procedures: the totally endoscopic isolated tricuspid valve surgery (EITVS) group (16 patients); and the TTVR group (5 patients). Inclusion criteria were as follows: (I) previous LSVS and secondary severe TR; (II) underwent totally EITVS or TTVR; (III) presence of diuretic refractory symptoms. The exclusion criteria were: (I) concomitant valve surgery or other procedures; (II) severe TR due to endocarditis, congenital disease, or trauma; (III) prostheses failure requiring repeat tricuspid valve replacement (TVR); (IV) severe pulmonary hypertension, significant right ventricle systolic dysfunction or right heart failure (RHF) detected by right heart catheterization.

### 2.2. Endpoints and definition

The primary endpoint was the procedure success rate of minimally invasive ITVR, which consisted of in-hospital mortality and residual TR > grade 1+. In-hospital mortality was defined as all-cause mortality within 30 days of the procedure regardless of discharge. Grading criteria of TR were as follows: 0 for none, 1+ for mild, 2+ for moderate, 3+ for moderate-to-severe, 4+ for severe, and 5+ for massive. The secondary endpoints were major morbidity and minor complications during hospitalization and follow-up. Major morbidity included permanent stroke, renal failure, prolonged ventilation over 24 h, and reoperation for any reasons (9). Other complications included pneumonia, fistula, wound infections, hemorrhage, chyle leakage, and other surgical complications that cause prolonged length of stay and readmissions.

### 2.3. Surgical procedure

All operations were performed according to a standard procedure and completed on the beating heart. The surgical techniques for endoscopic isolated tricuspid valve valvuloplasty (TVP) were previously described (10). In brief, after induction of general anesthesia, bilateral jugular cannulation was performed by our anesthesia. Then, the right femoral artery was cannulated followed by cardiopulmonary bypass (CPB) establishment. The working port, auxiliary port, and thoracoscopic port were positioned in the 4th, 4th, and 5th intercostal spaces, respectively. The pericardium and right atrial wall were incised to explore tricuspid valve. Valvuloplasty procedures including ring annuloplasty, edge-to-edge, papillary muscle plasty, artificial chordae, bicuspidization, and cleft closure were performed as needed. In cases of tricuspid leaflet tethering, most common in patients with rheumatic heart disease, leaflet patch (bovine pericardial, anti-calcification bovine pericardial, or Gore-Tex patch) augmentation was preferred (11). Transesophageal echocardiography (TEE) was performed to confirm the effect of valvuloplasty. TVR is considered when there are severe valve lesions, calcification, extreme annular dilatation, severe fibrosis of the subvalvular apparatus, or failure of the TVP.

**FIGURE 1:**
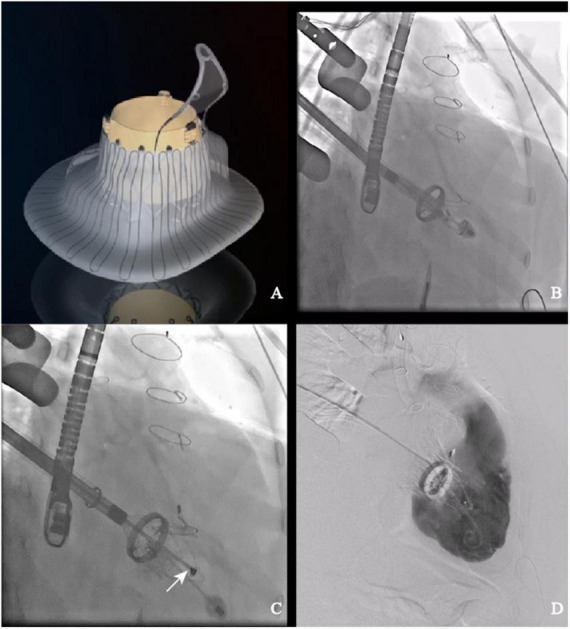
LuX-Valve and its application in interventional procedure. **(A)** Schematic of LuX-Valve; **(B)** coaxiality adjustment; **(C)** anchoring needle (white arrow) release; **(D)** post-implantation.

LuX-Valve (Figure 1A) is a novel TTVR valve delivered through a minimally invasive right thoracotomy. An approximately 8 cm incision was made in the 5th intercostal space, where the skin, pectoralis major and intercostal muscles were transected layer by layer. After systemic heparinization, the femoral vein was pierced with a 6F arterial sheath, and then cannulated with an angiography catheter. A purse-string suture was placed, and a pigtail catheter was advanced into the right ventricle. Both TEE and right ventriculography were performed to confirm the severity of TR. Pressure of right ventricle, pulmonary artery and right atrium were detected and recorded. Following the subtraction angiography guidance, the LuX-Valve delivery system was inserted from right atrium to right ventricle. The coaxiality between the delivery system and the tricuspid annular plane was adjusted until they were perpendicular to each other (Figure 1B). The anterior leaflet graspers were gradually released to grasp the anterior leaflet. Next, the atrial disc was released. Under the guidance of TEE, the correct orientation and position of the prosthetic valve were adjusted until minimize paravalvular leakage. Finally, the anchoring needle (Figure 1C) was released and nailed into the ventricular septum. The incision was closed after the delivery system was withdrawn. Right ventricular angiography confirmed the effect of the prosthetic valve and again recorded the aforementioned pressure (Figure 1D).

### 2.4. Follow-up

All enrolled patients completed follow-up. The follow-up aimed to record improvement in signs and symptoms, cardiac and valvular function, and any adverse events, including death, stroke, permanent pacemaker implantation (PPI), and heart failure-related hospitalization. Follow-up was conducted by telephone, and outpatient clinic. At each outpatient clinic visit, electrocardiogram and transthoracic echocardiography (TTE) were routinely performed. The follow-up ended on 20 August 2022.

### 2.5. Statistical analysis

SPSS statistical software (SPSS statistics 26.0) and R software (version 3.5.5) were used for statistical analyses. Continuous variables that conformed to a normal distribution were presented as mean ± standard deviation (M ± SD), and Student’s *t*-test was used to compare differences between two groups. Continuous variables that did not conform to a normal distribution were indicated by median (first and third quartiles), where Mann–Whitney U tests or Wilcoxon rank sum tests was used for comparison of two groups. Comparisons of variables between baseline and postoperative parameters were performed using paired *t* test or Wilcoxon test as appropriate. The counting data were represented by *n* (%), and inter-group comparison was assessed by Fisher exact test.

## 3. Results

### 3.1. Baseline characteristics

The baseline characteristics of 21 included patients are presented in Table 1. The overall mean age was (60.0 ± 8.4) years, and 15 (71.4%) were female. All patients had previously undergone LSVS with or without concomitant TVP. An average of 15.1 ± 6.2 years had elapsed since the last LSVS. Expect for severe TR, enlarged right atrium (100%) and ventricle (100%), and atrial fibrillation (85.7%) were most common seen. In total, 13 (61.9%) of the patients were in New York Heart Association (NYHA) Class III, and none were in NYHA Class IV. TTE demonstrated a mean fractional area change (FAC) (40.7 ± 2.8%), with a tricuspid annular plane systolic excursion (TAPSE) (14.4 ± 2.8 mm). Compared with the EITVS group, patients in the TTVR group were elder with worse NYHA functional class and cardiac status, thus they presented with higher clinical risk score (CRS) (12) [8.00 (8.00, 9.00) vs. 5.00 (3.25, 5.00), *P* = 0.001]. Other characteristics between two groups were similar without significant difference.

**TABLE 1:** Baseline characteristics.

Characteristics	Total (*n* = 21)	EITVS (*n* = 16)	TTVR (*n* = 5)	*P*-value
Age, year	60.0 ± 8.4	59.5 ± 8.8	61.6 ± 7.7	0.638
Female, *n* (%)	15 (71.4%)	11 (68.8%)	4 (80.0%)	1
BMI, kg/m^2^	22.3 ± 2.9	22.3 ± 3.0	22.1 ± 2.8	0.893
BSA, m^2^	1.6 ± 0.1	1.6 ± 0.1	1.6 ± 0.2	0.885
NYHA Class III, *n* (%)	13 (61.9%)	8 (50.0%)	5 (100%)	0.111
Urgent, *n* (%)	3 (14.3%)	0	3 (60.0%)	0.008
EuroScore II, %	4.11 (3.35, 5.14)	3.84 (3.35, 4.78)	4.54 (4.26, 7.00)	0.057
CRS, %	5.00 (4.00, 7.00)	5.00 (3.25, 5.00)	8.00 (8.00, 9.00)	0.001
**Comorbidities, *n* (%)**				
Atrial fibrillation	18 (85.7%)	13 (81.3%)	5 (100.0%)	0.549
Hypertension	4 (19.0%)	4 (25.0%)	0	0.278
Diabetes mellitus	9 (42.9%)	8 (50.0%)	1 (20.0%)	0.338
Coronary heart disease	3 (14.3%)	1 (6.3%)	2 (40.0%)	0.128
COPD	4 (19.0%)	2 (12.5%)	2 (40.0%)	0.228
Remote cerebrovascular disorder	2 (9.5%)	1 (6.3%)	1 (20.0%)	0.429
Cardiac pacemaker	1 (4.8%)	1 (6.3%)	0	1
NT-proBNP, pg/ml	569.1 (231.6, 913.2)	689.0 (230.8, 1,080.8)	456.3 (301.1, 654.6)	0.509
Serum creatinine, μmol/L	62.9 ± 13.7	63.7 ± 12.6	60.2 ± 18.3	0.632
Albumin, g/L	40.8 (39.3, 41.8)	39.5 (38.4, 41.1)	41.5 (40.6, 43.3)	0.057
ALT, U/L	17.0 (14.0, 21.5)	17.0 (14.0, 24.3)	16.0 (11.0, 20.0)	0.590
ESR, mm/h	28.8 ± 14.4	26.5 ± 16.1	34.0 ± 9.0	0.350
**Echocardiography**				
TR ≥ 5+, *n* (%)	7 (33.3%)	6 (37.5%)	1 (20.0%)	0.624
MR, *n* (%)	3 (14.3%)	2 (12.5%)	1 (20.0%)	1
AR, *n* (%)	9 (42.9%)	7 (43.8%)	2 (40.0%)	1
RAD, mm	77.7 ± 16.9	77.7 ± 19.3	77.8 ± 6.8	0.990
RVD, mm	58.4 ± 5.8	59.0 ± 6.0	56.6 ± 5.3	0.437
LVEF, %	61.9 ± 6.8	62.1 ± 7.5	61.2 ± 3.9	0.811
PASP, mmHg	38.9 ± 7.4	39.1 ± 6.4	38.2 ± 11.1	0.827
FAC, %	40.7 ± 2.8	40.3 ± 2.7	41.4 ± 3.1	0.492
TAPSE, mm	14.4 ± 2.8	13.7 ± 2.9	15.9 ± 2.1	0.222
Surgery time interval, year	15.1 ± 6.2	14.9 ± 6.9	15.8 ± 3.3	0.792
**Previous valve surgery, *n* (%)**				
AVR	1 (4.8%)	1 (6.3%)	0	1
MVR	6 (28.6%)	3 (18.8%)	3 (60.0%)	0.115
DVR	4 (19.0%)	4 (25.0%)	0	0.532
MVR + TVP	7 (33.3%)	6 (37.5%)	1 (20.0%)	0.624
DVR + TVP	2 (9.5%)	1 (6.3%)	1 (20.0%)	0.429
Bentall	1 (4.8%)	1 (6.3%)	0	1

BMI, body mass index; BSA, body surface area; CRS, clinical risk score; COPD, chronic obstructive pulmonary disease; ALT, alanine aminotransferase; ESR, erythrocyte sedimentation rate; TR, tricuspid regurgitation; MR, mitral regurgitation; AR, aortic regurgitation; RAD, right atrial diameter; RVD, right ventricle diameter; LVEF, left ventricular ejection fraction; PASP, pulmonary artery systolic pressure; FAC, fractional area change; TAPSE, tricuspid annular plane systolic excursion; AVR, aortic valve replacement; MVR, mitral valve replacement; DVR, double valve replacement; TVP, tricuspid valvuloplasty.

### 3.2. Surgical results

**FIGURE 2:**
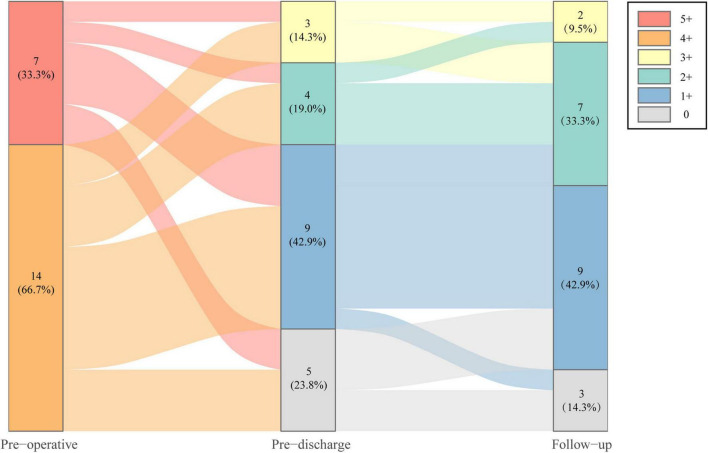
Severity change of tricuspid regurgitation of study population at preoperative, pre-discharge, and follow-up.

Operative data and postoperative outcomes are summarized in Table 2. The minimally invasive ITVR procedures were 100% successful in all patients without any perioperative mortality, sternotomy conversion, or reoperation. In the EITVS group, 8 (50.0%) patients had leaflet patch augmentation; 2 (12.5%) patients underwent TVR. Postoperative TR severity (Figure 2) improved significantly in two groups (both *P* < 0.001)—in the EITVS group, grade 3+, 2+, 1+, and 0 TR occurred in 3 (18.8%), 4 (25.0%), 7 (43.8%), and 2 (12.5%) patients, respectively; in the TTVR group, 3 (60.0%) patients had no TR while 2 (40.0%) patients had grade 1+ TR. The length of intensive care unit (ICU) stay was 1.8 (1.7, 5.6) days. Five (31.3%) patients in the EITVS group had prolonged mechanical ventilation, 3 of whom were combined with pneumonia. One (20.0%) patient in TTVR group developed prosthetic valve thrombosis. All patients were successfully discharged.

**TABLE 2:** Surgical outcomes.

Variables	Total (*n* = 21)	EITVS (*n* = 16)	TTVR (*n* = 5)	*P*-value
CPB time, min	–	111.38 ± 35.9	–	–
Convert to sternotomy, %	0	0	0	–
Perioperative mortality, %	0	0	0	–
Reoperation, %	0	0	0	–
**Valvuloplasty technique, *n* (%)**				
leaflet patch augmentation	–	8 (50.0%)	–	–
tricuspid annuloplasty ring	–	14 (87.5%)	–	–
Artificial chordae	–	3 (18.8%)	–	–
RAV reduction	–	2 (12.5%)	–	–
ICU stay, days	1.8 (1.7, 5.6)	1.9 (1.6, 6.1)	1.8 (1.2, 11.4)	0.620
Prolonged ventilation, *n* (%)	5 (31.3%)	5 (31.3%)	0	0.278
Pneumonia, *n* (%)	4 (19.0%)	3 (18.8%)	1 (20.0%)	1
Stroke, *n* (%)	0	0	0	–
Renal failure, *n* (%)	0	0	0	–

CPB, cardiopulmonary bypass; RAV, right atrium volume; ICU, intensive care unit.

### 3.3. Follow-up results

**FIGURE 3:**
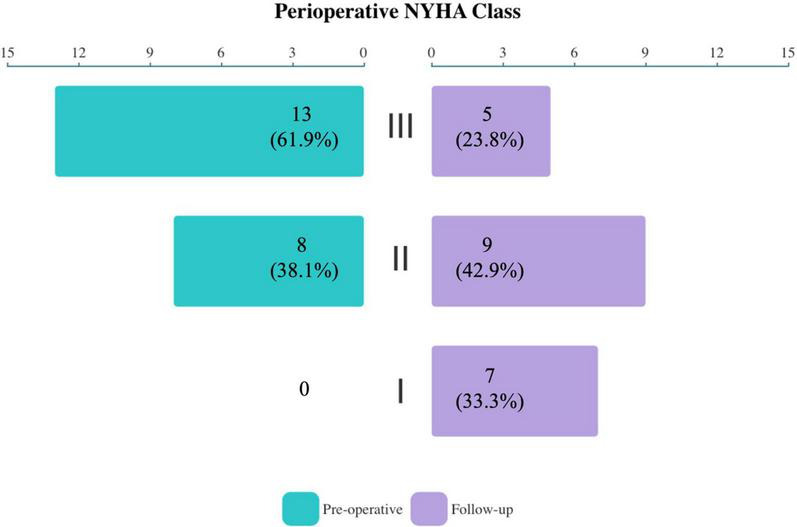
Compared with baseline, the overall NYHA functional class of study population significantly improved at follow-up (*P* = 0.004).

During the median follow-up of 16.8 months (IQR, 13.0–20.6 months), TR severity (Figure 2) was still significantly improved in both groups compared with baseline (both *P* < 0.001). In the EITVS group, the TR grade further decreased in 2 (12.5%) patients, and 5 (31.3%) patients showed deterioration from TR, which causes two patients remained in grade 3+. The TR grade in the TTVR group was maintained at 2 (40.0%) patients with grade 1+ due to paravalvular leak, and 3 (60.0%) patients with grade 0. The TTVR group had a higher success rate in reducing TR to ≤ grade 1+ than the EITVS group (100 vs. 43.8%, *P* = 0.045). Overall right atrial diameter (77.7 ± 16.9 vs. 65.4 ± 12.1 mm) showed significantly reduction compared with baseline (*P* < 0.001). By warfarin treatment, the valvular thrombosis in the TTVR group was negative at TTE follow-up at 3 months. None of the patients developed severe symptoms or required reoperation. NYHA Class (Figure 3) significantly improved from baseline (*P* = 0.004).

## 4. Discussion

More than 22% of TR were secondary to LSVS, which is not uncommon (13). With the progression of TR deterioration and dilation of right atrium and ventricle, RHF occurs and is manifested by a variety of signs and symptoms, such as chest tightness, palpitations, dyspnea, even edema, pulmonary hypertension, hepatomegaly, and gastrointestinal bleeding. Patients with rheumatic heart disease were associated with higher rate of occurrence of secondary severe TR as disease or impaired right heart function progressed. Despite the definite efficacy of diuretic in RHF, their effect is transient and is unavailable to stop or reverse the progression of RHF. Indeed, both the European Society of Cardiology and the European Association for Cardio-Thoracic Surgery (ESC/EACTS) and the American Heart Association/American College of Cardiology (AHA/ACC) guidelines recommend the ITVR for severe TR after LSVS when feasible (14, 15).

Previous studies have reported that ITVR is challenging due to the reoperation associated with a mortality rate of ≥ 10% (16, 17). The use of minimally invasive approach, such as right thoracotomy, and endoscopy in ITVR is one way to improve the short- and long-term clinical prognosis (18–20). In our study, the early outcomes were satisfactory, showing a 100% success rate without any mortality or reoperation. These results were mainly attributed to the following factors: minimally invasive access, patient selection, and perfect surgical timing. The totally endoscopic TVP or TVR performed on the beating-heart is not only provides less trauma, but also shortens the surgical and ICU stay times. However, it remains controversial whether TVP or TVR is the best strategy for ITVR. With the continued advances in technology and materials of TVP, it generally performs well in reducing TR and improving cardiac function, as well as the quality of life. More importantly, multiple studies have shown that TVP is associated with lower complication rates and all-cause mortality compared with TVR, therefore TVP has become the prior strategy in various centers (20–23). However, there are concerns about TR recurrent after TVP from a long-term perspective. Chen et al. (24, 25) recommended TVR using bioprosthetic valves, which provided comparable results in long-term follow-up. Besides, the mechanism of ITVR is complex. Apart from functional TR, some ITVR manifest as extreme enlargement of right ventricle or/and atrium, coaptation significantly lower than annular level with annular dilatation and, severe leaflet damage, making TVR a simple and feasible option. Based on the results of our single-center experience, we prefer TVP to TVR because TVP is essential to prevent valvular obstruction and prosthetic valve failure, which would reduce complications of bleeding, embolism, atrioventricular block, and a third-time operation (10, 19). Besides, TVR usually drops substantial TR from severe to none or mild grade, which could greatly increase the preload of the right heart postoperatively and worsen rather than improve its ejection function. This may explain why TVP is more beneficial in terms of 30-day mortality and cardiovascular events (10, 23). We also recommend applying multiple valvuloplasty techniques in TVP–for example, leaflet augmentation to enlarge the tethering valve and enhance its coaptation; prosthetic ring annuloplasty to restore annular dilation. As for LuX-Valve, it is a radial force–independent TTVR device. The advantages of TTVR are not limited to fewer incisions independent of CPB. First, the size of the implanted LuX-Valve does not traditionally depend on the tricuspid annulus size but on the effective orifice area, thus avoiding the negative effects on right ventricular function and complications such as PPI. Second, the sealing skirts design is aimed to prevent paravalvular leak. As the right heart volume and tricuspid annulus gradually narrow postoperatively, the prosthetic valve would be further adhered. Last but not least, the interventricular septal anchor and graspers are able to prevent malpositioning of the valve. These features allow LuX-Valve to be effectively applied to the treatment of multiple etiologies of TR.

Studies have shown that the mortality rate of ITVR are varies (19, 26, 27). In addition to surgical approach evolution, patient selection and surgical timing are predominating factors, suggesting that the low mortality rate might relate to worse cardiac status and late timing of surgical intervention. The timing of ITVR is closely coupled to preoperative status and right heart function. In this study, although the CRS was 5.00 (4.00, 7.00) predicting a mortality and morbidity risk ranging from 19 to 59%, most patients were in NYHA Class III, and had normal nutritional status without hepatic and renal failure. Before surgery, patients were routinely administered diuretic therapy, and then the right heart function was assessed by TTE. Currently, it lacks recognized and objective criteria for right hear function grading. FAC, TAPSE, Tei index, and DTI-derived tricuspid lateral annular systolic velocity are parameters easily assessed by TTE for quantification of right ventricle function (28, 29). We use FAC and TAPSE to select patients in this study. All patients had FAC (40.7 ± 2.8%) greater than 35%, with TAPSE (14.4 ± 2.8 mm) closed to 15 mm, indicating that they were at an early stage of RHF. EITVS was generally prohibited if severe pulmonary artery systolic pressure (PASP) (> 55 mmHg) persisted after pharmacological treatments, but TTVR might be more appropriate for such high-risk patients based on our study results. Compared with the EITVS group, the TTVR group had higher CRS [8.00 (8.00, 9.00) vs. 5.00 (3.25, 5.00), *P* = 0.001], but a higher success rate in reducing TR to less than grade 1+ (100 vs. 43.8%, *P* = 0.045) at follow-up. In addition, 2 (12.5%) patients in the EITVS group had grade 3+ TR in the short-term, which is in line with previous study (30). This may be attributed to inadequate valvuloplasty techniques (only annuloplasty ring) and patch detachment. Although their right heart dimensions and symptoms improved after reducing 1 to 2 grade of TR, it is not known whether progression from TR requires a third operation in the long term.

Our study certainly has some significant limitations, particularly due to its retrospective nature and small sample size. As a single-center cohort study, the population of severe TR after LSVS is small, and some of patients prefer conservative treatment rather than reoperation. Another limitation is the heterogeneity of the patient cohort. Patients had different duration of pharmacological treatments before surgery, which might have an impact on surgical outcomes. Further studies are required to compare EITVS and TTVR for patients at equivalent high risk. However, to our knowledge, our cohort is the first to present ITVR using a minimally invasive technique through endoscopic and transcatheter approaches. The long-term effects of the two interventions also await further investigation.

## 5. Conclusion

In our series, the early outcomes of minimally invasive ITVR, including EITVS and TTVR, are excellent in selected patients, with zero operative mortality. TTVR is a reliable alternative for patients with high surgical risk. To improve the results of ITVR, it’s necessary to improve patient’s preoperative status or perform reoperation before the onset of significant RHF. Further studies with a larger sample size and a longer follow-up period are awaited.

## Data availability statement

The original contributions presented in this study are included in this article/supplementary material, further inquiries can be directed to the corresponding authors.

## Ethics statement

The studies involving human participants were reviewed and approved by the Ethics Committee of Guangdong Provincial People’s Hospital. The patients/participants provided their written informed consent to participate in this study.

## Author contributions

JL and TT were involved in the conception and design of the study. TT, WG, XZ, HW, and HL were contributed to data collection and analysis. JL, TT, HH, and JM were involved in the construction of the manuscript. JL, HH, JZ, and JC were critically revised the manuscript for intellectual content. HG was responsible for full-text proofreading. All authors read and approved the final manuscript.
